# Uncovering the Hidden Dangers and Molecular Mechanisms of Excess Folate: A Narrative Review

**DOI:** 10.3390/nu15214699

**Published:** 2023-11-06

**Authors:** Ali M. Fardous, Ahmad R. Heydari

**Affiliations:** 1 Department of Nutrition and Food Science, Wayne State University, Detroit, MI 48202, USA; ali.fardous@wayne.edu; 2 Barbara Ann Karmanos Cancer Institute, Wayne State University, Detroit, MI 48202, USA

**Keywords:** unmetabolized folic acid (UMFA), folic acid, one-carbon cycle, methylation, epigenetic, carcinogenesis, aging, neurodevelopment, embryogenesis, folate

## Abstract

This review delves into the intricate relationship between excess folate (vitamin B9) intake, especially its synthetic form, namely, folic acid, and its implications on health and disease. While folate plays a pivotal role in the one-carbon cycle, which is essential for DNA synthesis, repair, and methylation, concerns arise about its excessive intake. The literature underscores potential deleterious effects, such as an increased risk of carcinogenesis; disruption in DNA methylation; and impacts on embryogenesis, pregnancy outcomes, neurodevelopment, and disease risk. Notably, these consequences stretch beyond the immediate effects, potentially influencing future generations through epigenetic reprogramming. The molecular mechanisms underlying these effects were examined, including altered one-carbon metabolism, the accumulation of unmetabolized folic acid, vitamin-B12-dependent mechanisms, altered methylation patterns, and interactions with critical receptors and signaling pathways. Furthermore, differences in the effects and mechanisms mediated by folic acid compared with natural folate are highlighted. Given the widespread folic acid supplementation, it is imperative to further research its optimal intake levels and the molecular pathways impacted by its excessive intake, ensuring the health and well-being of the global population.

## 1. Introduction

It is estimated that over a third of the population in North America is chronically exposed to high levels of dietary folate from a combination of voluntary supplementation and food fortification with folic acid (FA) [1,2,3]. Eukaryotic cells lack the capability to synthesize folate de novo, establishing folate as an essential nutrient for higher organisms and humans. While the benefits of adequate folate intake are well documented, there is mounting evidence linking its excess with deleterious effects on human health by impacting cancer, immunity, birth outcomes, cardiovascular disease, and overall mortality. The association between excess folate and these negative outcomes is well-studied, but the causative mechanisms are not. Understanding and unraveling the mechanisms by which excess folate from natural sources, supplementation, enrichment, and fortification of the food supply could negatively impact health and disease is of critical importance. Recent reports focused on the areas of nutrition, folate metabolism, cancer, and aging, with each concluding that there are substantial knowledge gaps relating to the effects of excess folate on human health [4,5,6].

A comprehensive narrative on the potential impacts of excessive folate intake on health and disease is currently lacking. Additionally, there is no consensus regarding the potentially harmful role of excess folate supplementation, and there is a need to reconcile conflicting reports to identify gaps and areas requiring further research. This review aimed to provide a synthesis of the existing literature describing the potential effects of excess folate on health and discuss the possible molecular mechanisms underlying these effects, thus contributing to a better understanding of the complex relationship between folate intake and human health.

## 2. Methods

PubMed and Web of science databases were searched using permutations of the terms “excess folate”, “high folate”, “folic acid”, “UMFA”, in combination with “pregnancy”, “carcinogenesis”, “methylation”, “embryogenesis”, “neurodevelopment”, “autism”, and “mortality”. The reviewers screened the results using the following inclusion criteria: availability as full text in English; published between January 2000 and June 2023; and categorized as original research, a review, a systematic review, a meta-analysis, or a letter to the editor. Titles and abstracts were screened for the above criteria and relevance to the search topic. Articles that did not meet the specified criteria, as well as articles that did not report folate intake, supplement dosage, or physiological levels, were excluded. Additional literature was obtained from the references of relevant manuscripts (snowball method).

## 3. Results

The field of study encompassing folate research is large and rapidly evolving. Careful analysis of the existing literature highlighted key considerations, characterizations, and definitions that are addressed herein. Additionally, the literature revealed several health outcomes impacted by excess folate, which were categorized into the following seven classifications: pregnancy outcomes, disease risk in offspring, neurodevelopment, immune function and allergies, carcinogenesis, overall mortality, and other health effects. Each category is further elaborated through a detailed discussion of the relevant literature by addressing human studies conducted in that area, as well as animal cell culture studies where applicable. This was followed by elucidating the identified molecular mechanisms implicated in the effects of excess folate in the subsequent section. These mechanisms were grouped into two broad categories encompassing one-carbon-cycle-dependent and -independent mechanisms. The mechanistic findings of individual studies were examined, synthesized, and subcategorized within this provided mechanistic framework.

## 4. Discussion

### 4.1. Key Considerations Concerning Dietary Folate Intake

#### 4.1.1. Folic Acid versus Natural Folate

The distinction between natural folate and synthetic FA is an important consideration when evaluating the potential risks of excess intake. Natural folate, mainly in the form of 5-methyl-tetrahydrofolate (5-m-THF) and, to a lesser extent, 5-formyl-tetrahydrofolate (5-f-THF), are biologically active forms that do not necessitate activation by dihydrofolate reductase (DHFR) as folic acid does. Natural folate intake is constrained by the limited amount present in food sources, as well as its lower bioavailability and stability. In contrast, large doses of synthetic FA can be easily consumed through food fortification and supplementation, making folic acid the primary contributor to elevated folate pools in the human population. Limited research has been conducted using physiologically relevant levels of natural folate in the place of FA. Natural folates are inherently less stable and bioavailable than FA, which creates technical and logistical challenges for their integration into basic research. Additionally, the widespread use of the terms “folate” and “folic acid” interchangeably has led to the misconception that these forms are equivalent. In this review, “folate” refers to all folate species, synthetic and natural, that are found physiologically and in foods, while “folic acid” denotes the fully oxidized synthetic folate used in supplementation and food fortification.

#### 4.1.2. Folic Acid Intake

Folic acid is used almost universally in fortification and supplementation, as well as in conducting most laboratory research. Women of childbearing age taking prenatal vitamins, as well as children and adults taking vitamins high in FA, are likely to exceed the age-adjusted tolerable upper intake level (UL) of folate intake due to a combination of food fortification and supplementation [4,7]. FA supplementation is linked to elevated circulating unmetabolized folic acid (UMFA) in a dose-dependent manner [8]. A growing body of literature linking excess FA and UMFA to potential adverse health outcomes exists. However, evidence of causality between excess FA, UMFA, and various adverse effects remains inconclusive [5].

#### 4.1.3. Prevalence of Elevated Folate Levels in the North American Population

Mandatory fortification in combination with supplementation with FA led to a more than doubling of the USA population’s serum folate concentration in the last thirty years [9,10,11]. In the USA, up to 35% of the population is taking folic-acid-containing supplements and roughly 5 percent of the population exceeds the UL of folate intake. The average folate intake is estimated to be 813 µg/day for men and 724 µg/day for women [1]. The Canadian Health Measures Survey identified that up to 40% of participants had high folate levels (defined as RBC folate > 1360 nmol/L) [2,3,12]. Unmetabolized folic acid was detected in over 95% of Americans participating in the National Health and Nutrition Examination Survey (NHANES), with significantly higher UMFA levels recorded among supplement users and older individuals [9,13]. Similarly, high levels of FA and UMFA were found in pregnant Canadian women and umbilical cord blood, with over 97% of pregnant Canadian women having detectable plasma UMFA [14,15] and up to 26% of pregnant women exceeding the UL of FA [16].

#### 4.1.4. Defining Excess Folate

A significant gap exists in the understanding of what constitutes excess folate in humans and research models. Determining the levels at which folate intake becomes excessive is an elusive task since there is a lack of dose–response data and comprehensive documentation of adverse effects at a high folate intake has not been established [4]. The recommended daily allowance (RDA) of folic acid varies depending on age, sex, and life stage. Adult men and women are recommended to intake 400 µg of dietary folate equivalent (DFE), increasing to 500 µg and 600 µg DFE for breastfeeding and pregnant women, respectively. The tolerable upper intake level (UL) for folic acid was set at 1000 µg per day for adults. However, the determination of folic acid UL relied on limited and low-quality observational case study data [17]. Generally, folate exposure at dosages exceeding the adult UL is considered excessive. However, this criterion does not consider chronic exposure to elevated folate intake that approaches the UL and the inherent variability and mutations in folate cycle enzymes that are prevalent in humans [18,19].

In rodent studies, excess folate can be defined as exceeding the accepted standard baseline supplementation of 2 mg/kg diet. However, the optimal folate requirements for animals may be lower, and the activity of rate-limiting one-carbon metabolism enzymes is typically much higher in rodents compared with humans [20,21,22,23]. Chow diets fed to laboratory rodents can contain a variable amount of FA ranging from 2 to 15 mg/kg diet and averaging about 8 mg/kg diet [22]. The reason for supplementing rodent diets with excessive amounts of FA is not clear. However, it is likely that suppliers of research animals and their diets defaulted to higher levels of folate supplementation to ensure rapid growth and large litter sizes.

In cell culture studies, cells and tissues are subjected to elevated levels of folates that greatly exceed the physiological levels found in tissues to support growth and proliferation. In human plasma, the physiological levels of natural folate range from 150 to 450 nM, while standard cell culture media contains 2200 nM (RPMI) or 9000 nM (DMEM) of the non-physiological synthetic folic acid [24,25]. This implies that most studies utilizing standard cell culture conditions could be confounded by these supraphysiological concentrations [25,26].

### 4.2. Health Outcomes of Excess Folate

#### 4.2.1. Pregnancy Outcomes

##### Prenatal Folate Intake and Neural Tube Defects

The primary goal of the folic acid food fortification mandates that were instituted in various countries around the world is to reduce the incidence of births with neural tube defects (NTDs). The folate fortification mandate has been notably successful in reducing the incidence of NTDs in populations where it has been implemented. Folate adequacy for preventing NTDs is critical in the first 4 weeks of gestation. Folate supplementation in the following weeks, postnatally, and during breastfeeding could potentially provide benefits to maternal and fetal health with varying and inconsistent outcomes [27]. Supplementation at the WHO recommended levels of 400 µg/day in the periconceptual period protects against NTDs, with largely positive health outcomes reported. However, FA intake at higher doses, especially when approaching or surpassing the upper intake limit and administered for extended durations, is at times associated with deleterious pregnancy and offspring outcomes. A detailed analysis of the effects of FA supplementation on gestation and long-term health was comprehensively reviewed elsewhere by Silva et al. [27]. Common prenatal vitamins typically include an FA dose of 1 mg/day with some prescribed regimens using doses of up to 5 mg/day. On average, women taking prenatal vitamins are likely to surpass the UL [28], leading to elevated levels of total folate and UMFA in maternal and fetal samples [29,30].

##### Prenatal Folate Intake and Non-NTD Outcomes

The impact of FA supplementation and specifically high-dose FA on non-NTD pregnancy outcomes is not clear [27]. The link between FA supplementation and oral cleft incidence is inconsistent with studies reporting a protective effect [31,32,33,34], no clear association [35,36], or an increase in oral cleft births [37]. In one study, high FA (4 mg/day) did not compromise fetal growth or provide additional protection against orofacial clefts compared with 0.4 mg/day [35]. Another study that used the same dose of 4 mg FA/day in early pregnancy saw increased maternal UMFA and serum folate, with no significant effect on RBC folate or evidence of altered one-carbon metabolism [38]. A Cochrane Review found no conclusive evidence that FA supplementation improved focused pregnancy outcomes, such as preterm birth, stillbirth, anemia, serum, and RBC folate levels [39]. In a Greek cohort, 5 mg FA/day was found to be protective against preterm birth and low birth weight [40]. FA supplementation provided benefits in reducing the incidence of congenital heart defects, irrespective of the timing and dosage during pregnancy, with beneficial association attributed to a high dose of FA (5 and 6 mg/day) [41,42]. In a Spanish cohort where pregnant women were supplemented with FA in doses exceeding 1 mg/day, an association was found with decreased birth length and an increase in the risk of low birth weight [43]. Increased incidence of position plagiocephaly was identified in a nested case–control study performed in the Netherlands in correlation with periconceptional high doses of FA [44].

##### Gestational Diabetes Mellitus

High folate concentration was linked to an increased risk of gestational diabetes mellitus (GDM) in Chinese cohorts [45,46]. Specifically, a higher FA dose, as well as the timing and duration of the supplementation, was associated with an increased risk for the development of GDM [47,48,49,50], with a significant GDM association when high FA was combined with an imbalance in vitamin B12 levels [51].

#### 4.2.2. Disease Risk in Offspring

##### Folate Intake Modulates the Epigenome

Folate intake plays a critical role in the availability of the methyl donors necessary for epigenetic programming during conception, gestation, and early development. The paradigm that maternal exposure to various stressors, dietary factors, and nutrient excess or deficiency during the prenatal period and early development can modulate the risk for developing diseases later in life is well accepted [52]. As scientific knowledge advances, much of the etiology behind these effects has been attributed to epigenetic modifications and imprinting and is influenced by one-carbon metabolism [52,53,54]. Folate and its reduced intermediates play a crucial role in the one-carbon cycle and in regulating the processes involved in methylation and epigenetic modifications. Excess FA supplementation may potentially alter gene expression and lead to aberrant epigenetic programming in progeny [55,56]. Understanding the link between folate status in the parents, offspring, and epigenetic modifications can further our understanding of the origins of adult diseases [57,58,59].

##### Maternal Folate Intake and Transgenerational Disease Risk in Humans

Numerous human and animal studies identified associations between excess folate intake in mothers and disease risk in offspring, though the outcomes have been inconsistent. In the Indian subcontinent, where expecting mothers are often prescribed folic acid doses as high as 5 mg/day, high folate concentrations during pregnancy were linked to insulin resistance in children [60]. Results from a UK cohort of pregnant women found that FA supplementation after the 12th week of pregnancy impacts DNA methylation in the offspring, with significant changes in the methylation of regions that regulate insulin-like growth factor 2 in infants, potentially altering susceptibility to chronic diseases [57]. A systematic review by Xie et al. aimed at exploring the association between maternal folate status and the risk of obesity and insulin resistance in offspring could not reach a definitive conclusion due to inconsistencies in data collected from animal and human studies [61].

##### Paternal Folate Intake and Disease Risk in Progeny

The developmental origins of health and disease hypothesis suggest that fetal programming is influenced by maternal nutrition, among other factors. Moreover, growing evidence indicates that the paternal environment and diet significantly impact progeny’s health and risk of developing disease through epigenetic changes in sperm [62]. High doses of paternal folic acid supplementation were associated with increased gestational duration in births conceived through assisted reproduction [63]. Male offspring of mice fed a high-folate diet had a decreased sperm count, altered methylation in key genes, increased postnatal mortality in offspring, and reduced litter size [64]. Similarly, high-dose folic acid resulted in sperm DNA hypomethylation in both men and mice [65]. While moderate folic acid supplementation was beneficial in reducing some assisted reproduction-induced DNA methylation variance, high-dose supplementation produced deleterious effects with sex-specific outcomes [66].

##### Excess Folate Induces Significant Transgenerational Effects in Animal Studies

While the clinical significance of altered methylation in response to excess folate in humans is not clear, animal studies provided a more robust causative link between maternal folate intake, epigenetic changes, and disease risk. In rodents, maternal and post-weaning supplementation with FA significantly impacted global and gene-specific methylation in offspring [67], altered embryonic development [68] and cardiac gene expression [69], and modulated the expression of genes involved in growth [70]. Alterations of DNA methylation and imprinting patterns were partially responsible for deleterious transgenerational effects observed when either folate-deficient or excess-folate diets were fed to female mice [63]. Excessive FA during pregnancy induced obesity and altered the epigenetic profile of the offspring in rats [71,72], and led to metabolic dysfunction in late adulthood [73]. Similarly, other studies found that excess perinatal folic acid supplementation caused insulin resistance, dyslipidemia, and disrupted glucose metabolism and hepatic fat metabolism in both mice [66] and rat offspring [74,75,76].

#### 4.2.3. Neurodevelopment

##### Impact of Excess Prenatal Folate on Neurocognitive and Psychomotor Development

Excess folic acid supplementation during pregnancy is linked to both beneficial and detrimental effects on neurodevelopmental outcomes in children. Although FA supplementation is beneficial during the pre-conceptual period and early weeks of gestation by preventing neural tube defects, continued supplementation may significantly impact children’s neurodevelopment through epigenetic alterations [77,78]. FA supplementation at the WHO-recommended level of 400 µg/d beyond the first trimester was associated with benefits on neurocognitive development in children, with positive effects on cognitive development observed at ages 7 and 11 [79,80]. In a study where high-dose FA supplementation was implemented, cord blood analysis revealed significant changes in DNA methylation, exhibiting sex-specific and loci-specific alterations in genes related to neurodevelopment [81]. Doses exceeding the UL during the periconceptual phase were linked to impaired neurocognitive development in children [82,83]. High-dose FA (>5 mg/day) supplementation in pregnant mothers led to poor psychomotor development in their children compared with those receiving 0.4–1 mg/day [84]. In a small Greek cohort (*n* = 58), high-dose FA supplementation (5 mg/day) in early pregnancy improved children’s vocabulary development, verbal comprehension, and communication skills, while doses exceeding 5 mg/day showed no benefit [40].

##### Impact of Excess Prenatal Excess Folate on Autism Spectrum Disorders Risk

The relationship between FA supplementation and autism spectrum disorders (ASDs) is complex and marked by contradictory findings. Recent systematic reviews found inconclusive evidence associating FA supplementation and ASD [83]. However, some studies did find a significantly increased risk of ASD with elevated maternal plasma FA [83] and linked synthetic folic acid supplementation during pregnancy to an increased autism risk [85]. Some suggested that excess FA leading to unmetabolized folic acid (UMFA) can potentially heighten the ASD risk [86]. Moderate prenatal FA supplementation of at least 400 µg from the diet and supplements was associated with a decreased ASD risk in children [79,80], while excess FA corresponded with lower cognitive development and increased ASD risk [81,83,87]. This suggests a U-shaped relationship, where both inadequate and high FA exposure could increase the ASD risk [87].

##### Excess Prenatal FA Causes Significant Neurodevelopmental Defects in Rodents

In animal models, excess FA during the prenatal period is associated with an adverse impact on behavior, memory, embryonic growth, methyl metabolism, and neurodevelopment [88]. In rodent studies, excess maternal FA supplementation resulted in deleterious behavioral abnormalities in offspring, including anxiety-like behavior, hyperactivity, impaired reversal learning, and seizure susceptibility [83,87,89]. The collected evidence suggests that the influence of excess prenatal FA on the type of behavioral abnormalities is sex specific [70,90,91,92]. Both deficient and excess FA during pregnancy impaired cortical neurodevelopment in mice offspring [93]. In another study, moderate FA supplementation led to behavioral changes and sex-dependent alterations in one-carbon metabolism [94]. High-dose FA supplementation in mice caused neural tube defects and disrupted embryonic development [70,95,96]. Neurodevelopmental toxicity in male mouse offspring brains was observed when a 2.5-fold FA requirement was provided one week before mating and continued through pregnancy and lactation [97]. In rats, high FA doses administered periconceptually decreased the offspring seizure threshold [96], while high FA intake (20 mg/kg) impaired rat dam’s memory and Na+, K+—ATPase activity in the pup’s hippocampus [98].

#### 4.2.4. Immune Function and Allergies

##### Role of Folate in Immune Function

Folate plays a crucial role in maintaining a healthy immune system, influencing both innate and adaptive immune responses. Adequate folate status is essential for optimal immune function, while deficiency or excess of folate can lead to immune dysregulation. Folate is required for the production and maintenance of fast-proliferating white blood cells and is necessary for the maturation of lymphocytes. Folate plays a role in regulating inflammation and the production of pro-inflammatory cytokines [99]. Folate deficiency has been associated with impaired immune cell function and increased levels of tumor necrosis factor-alpha (TNF-α) and interleukin-6 (IL-6), which can contribute to impaired immunity, chronic inflammation, and the development of inflammatory diseases [100]. Considerable evidence exists associating folate intake with inflammation and autoimmune diseases [101,102,103].

##### Excess Folate and Dysregulation of the Immune System

Excessive folate intake can negatively impact innate immunity. In postmenopausal women, high levels of folic acid intake through supplements and a folate-rich diet were found to reduce NK cell cytotoxicity [104]. Unmetabolized folic acid (UMFA) was inversely associated with NK cell cytotoxicity in the same study [104].

The immune system plays a key role in regulating the onset and severity of airway inflammation and asthma, which is a disease characterized by an overactive immune response. Prenatal and periconceptual FA supplementation, and particularly excess FA, was associated with an increased risk of childhood asthma in several studies [55,105,106,107,108,109]. However, others could not find a clear association [110,111,112,113]. Late pregnancy FA supplementation, as well as the extended duration of the supplementation (over 6 months), was associated with the highest risk of childhood asthma [55,109]. Similarly, FA supplementation in the first trimester of pregnancy correlated with a higher probability and severity of bronchiolitis compared with non-supplemented mothers in the USA [114]. FA supplementation in pregnant women was associated with an increased risk of wheezing and lower respiratory tract infection in their offspring up to 18 months of age in a Norwegian cohort [115]. Others reported that high plasma folate in mid-pregnancy was linked to decreased odds of wheezing at three years of age [116]. In a recent systematic review and meta-analysis, Chen et al. concluded that FA intake can be a risk factor for respiratory tract allergies in infants and children; however, a significant association was found only for doses not exceeding 400 uμg/day, while the risk effect decreased with increasing FA dose. Moreover, the association between FA supplements and children’s risk of allergic diseases was significantly increased only in countries without FA fortification. This increase in risk was more pronounced in cases where mothers used folic acid supplements [117].

Atopic dermatitis is caused by the inflammatory response of an overactive immune system. A study reported that high folate and B12 levels during pregnancy were associated with a higher prevalence of atopic dermatitis in children [118], while third-trimester FA supplementation was associated with eczema in children but no other allergic outcomes [119].

A dysregulated immune system can trigger an inflammatory response that impacts multiple tissues and organs. In preclinical models, high-dose FA (50 mg/kg diet) administered intraperitoneally to a murine model of colitis improved associated inflammation [120]; however, others found that high-methyl-donor diets, including 5 mg/kg diet FA, increased susceptibility to colitis in offspring [121,122]. Similarly, excess FA supplementation in rat dams (20 mg/kg diet) exacerbated weight gain and inflammation [123]. High folic acid exposure negatively impacted human lymphocyte stress response, genomic stability, and DNA methylation in both in vitro and in vivo conditions [21,124]. Others found that folic acid supplementation could promote a proinflammatory transcriptomic milieu in the colon [125,126] and that excess FA increased inflammation in response to a high-fat diet in rats [127].

The impact of FA supplementation on inflammatory markers and associated diseases in humans remains inconclusive [128]. Further research is needed to elucidate the potential mechanisms underlying these associations and to determine the optimal FA supplementation strategies for minimizing immune-related risks.

#### 4.2.5. Carcinogenesis

Due to the importance of the folate cycle in DNA replication and repair, inadequate consumption of folate is considered an independent risk factor for cancer. A folate-deficient diet results in uracil misincorporation into DNA, leading to single- and double-stranded breaks [129]. On the other hand, excess folate may increase the cancer risk in the presence of preneoplastic lesions [17]. In rapidly proliferating pre-neoplastic or transformed cells, excess folate could help sustain high rates of growth by providing thymidylate and purines for DNA synthesis [130].

##### Folate and Colorectal Cancer: An Unsettled Debate

Colorectal cancer (CRC) is the second most prevalent cancer in Western societies, and it is most closely linked to folate intake. Following the fortification mandate, a small increase in the incidence of colorectal cancer in the USA and Canada was observed [131]. This association is considered controversial, as data from human and animal studies yielded contradictory results. Higher folate intake in natural and synthetic forms was associated with a decreased risk of CRC in women participating in the nurses’ health study [132]. Others found that vitamin B12 and folic acid supplementation significantly increased CRC incidence [131]. Long-term supplementation of FA at 1 mg/day was associated with increased multiplicity of colorectal adenomas, advanced lesions, and increased risk of prostate cancer in some studies [133,134,135]. However, in a recent systematic review, high folate intake was associated with reduced risk of colorectal cancer in the USA and Europe, particularly among people with moderate or high alcohol consumption [136].

##### Folate Intake and Breast Cancer Risk

Conflicting associations were found between folate intake and breast cancer risk [137,138,139]. In a meta-analysis by Chen et al., a U-shaped relationship was observed in prospective studies, where moderate folate intake (153–400 µg/day) decreased breast cancer risk compared with <153 µg/day, while an intake > 400 µg/day had no effect [138]. In another analysis by Zhang et al., moderate folate intake between 200–320 µg/day lowered the risk of breast cancer, while a significantly increased risk was observed with a daily folate intake exceeding 400 µg [137]. Recent studies concluded that a high intake of one-carbon-related vitamins, including folic acid, may be protective against estrogen-receptor-negative/progesterone-receptor-negative tumors and in individuals with moderate-to-high alcohol consumption [139,140].

##### Folate Intake Association with Other Cancers and Overall Cancer Risk

The association of folate intake with the incidence of non-CRC cancers is inconsistent and was reviewed in detail elsewhere [141]. Higher intake of folate was associated with a reduced risk of squamous cell carcinoma of the head and neck [142] and esophageal [143,144,145], oral [146], pancreatic [147,148], and bladder cancers [149,150]. We should note that the higher intake in these studies was unlikely to represent excess folate intake and the analysis was not stratified to isolate the effects of intake that approach or exceed the UL. No clear association between high folate intake and lung cancer was observed in a meta-analysis [151]. However, a study from Poland found that high systemic folate levels (RBC folate concentrations above 506.5 nmol/L) increased lung cancer risk in heavy smokers [152]. High intake of folate was not protective against ovarian cancer [153]. Despite an overall marginally negative association between endometrial cancer and folate intake, at higher intake levels ranging between 205 and 987 µg/day, cancer risk increased in a dose-dependent manner, suggesting a threshold effect [154]. In two randomized controlled trials, treatment with a moderate dose of FA and vitamin B12 caused an increased overall cancer incidence and mortality [142]. Similarly, a meta-analysis found a borderline significant increase in overall cancer risk with FA supplementation [155]. However, a more recent independent meta-analysis of RCTs did not find any significant effect of FA supplementation on overall cancer or colorectal cancer risk [156].

##### Folate in Preclinical Cancer Studies

Studies in rodents similarly revealed a complex and sometimes contradictory relationship between folic acid intake and tumorigenesis. The difference in models, timing, duration, dose, and experimental design led to inconsistent findings in rodents. The impact of folic acid supplementation on colorectal cancer in rodents was reviewed in detail elsewhere [157]. Notably, in some rodent studies that used high doses of folic acid (8 mg/kg), supplementation led to an increased occurrence of preneoplastic lesions and CRC [143,144,145]. Increased proliferation of colorectal epithelia and intake of folate after the development of neoplastic lesions was hypothesized to be responsible for the observed increased risk.

In an inducible breast cancer mouse model, a high-FA diet (10 mg/kg) significantly increased the tumor volume compared with baseline diets (2 mg/kg) [158]. Moderately high FA supplementation (5 mg FA/kg) prior to and during pregnancy and lactation in rats increased the risk and multiplicity of mammary adenocarcinomas in the offspring and reduced global DNA methylation [159]. In another rat model, FA supplementation (ranging from 5 to 10 mg/kg) promoted the progression of established mammary tumors [160].

In a chemically inducible prostatitis model, maternal folic acid supplementation at a dose of 2 mg/kg in rats led to a heightened incidence and severity of inflammation in their offspring [135]. This is especially relevant to the observation that folic acid supplementation was associated with an increased risk of prostate cancer in humans [161]. However, further research is needed to validate the effects and explore the underlying mechanisms.

In cell culture studies, FA depletion and supplementation enhanced the transformation of keratinocytes infected by the human papilloma virus [162,163]. Furthermore, depletion and over-supplementation of FA led to increased progression, migration, and invasion of the hepatocarcinoma HepG2 cells [164]. Similarly, in human lymphoblastoid cell lines, excess FA mimicked folate restriction by similarly impacting the oxidative stress response, DNA damage repair, and DNA methylation [124].

#### 4.2.6. Overall Mortality

Several studies sought to determine the relationship between high folate intake, chronic disease risk, and overall mortality. A systematic review by Colapinto et al. observed a negative association between high folate levels and adverse health outcomes. However, the variability in study methods, designs, and high folate cutoffs prevented definitive conclusions [12]. A large UK population cohort study (*n* = 115,664) found that higher dietary folate intake correlated with lower CVD risk and overall mortality [165], and a similar trend was noted in a recent NHANES survey data analysis [166]. Other studies, however, could not find a significant association between high folic acid intake and overall mortality [167,168]. A prospective analysis of 2011–2014 NHANES data found a higher mortality risk associated with increased serum folate levels, such as 5-m-THF, UMFA, non-methyl folate, and MeFox (a biologically inactive degradation product of 5-m-THF), as well as with 5-m-THF insufficiency [169]. Another cohort study based on 1999–2010 NHANES data reported modestly elevated all-cause and cardiovascular mortality in participants with high folate levels (>40 nmol/L) [170].

#### 4.2.7. Other Health Effects

Multiple studies offered insights into the relationship between folate intake and non-cancer health outcomes, particularly metabolic and cardiovascular diseases. A recent systematic review and meta-analysis found that folate supplementation improved fasting glucose, insulin resistance, and insulin levels, but had no effect on diabetes or HbA1c [171]. A large meta-analysis of randomized controlled trials could not establish a connection between folic acid supplementation and reduced cardiovascular risk, although a modest benefit was observed in stroke prevention [172]. However, high folate intake was associated with an increased risk of thyroid and endocrine disorders [5,172]. Studies in rats that compared the effects of 5-m-THF and FA supplementation showed that high doses of either form led to distinct adverse and harmful effects on metabolism [173]. Research that compared the effects of high intakes of natural and synthetic folate on health and disease is of particular significance, given that very few comparative studies have been conducted in human and animal models.

Folic acid supplementation is commonly used to treat hyperhomocysteinemia, which is a condition characterized by elevated homocysteine levels [174]. Homocysteine is a risk factor for stroke, cardiovascular disease, cognitive function, and adverse pregnancy outcomes [175,176,177,178]. In two separate studies, folic acid supplementations at 0.5 and 5 mg per day were similarly effective at reducing homocysteine levels [179,180]. A dose-finding interventional trial concluded that long-term doses as low as 0.2 mg of folic acid or lower effectively lowered homocysteine levels, with no further significant reduction observed at higher doses [181]. However, a high dose of folic acid (5 mg/day) for three months failed to normalize homocysteine levels in over 40% of participants in a recent study [182].

## 5. Molecular Mechanisms Underlying the Adverse Effects of Excess Folate Intake

**Figure 1:**
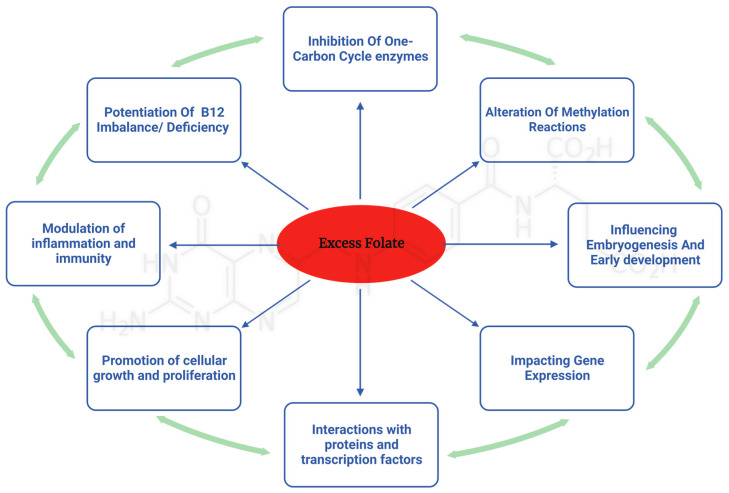
Implications of excess folic acid: impact on multifaceted pathways and their biological consequences.

While the previous sections outlined what is known about the association between excess folate intake and potentially deleterious health effects, the underlying mechanisms are not well defined. Isolating the responsible mechanisms is necessary for establishing causation. A diverse array of potential mechanisms from human and animal studies were identified in the literature. This section elaborates and discusses these potential molecular and genetic mechanisms that are hypothesized to mediate some of the potential deleterious effects of excess folate (Figure 1).

### 5.1. One-Carbon-Metabolism-Dependent Mechanisms

The one-carbon metabolism plays a crucial role in numerous cellular processes, including DNA synthesis, repair, and methylation. Excess folic acid can disrupt the balance of this metabolic cycle, leading to various adverse health effects. The mechanisms related to the one-carbon cycle can be further divided into the following categories:

#### 5.1.1. Vitamin-B12-Dependent Mechanisms

The aging population is particularly at risk for vitamin B12 deficiency, and there is concern that folic acid (FA) supplementation might mask and exacerbate the severe, potentially irreversible neurological damage caused by B12 deficiency [178,179,183,184]. Excess FA can conceal hematological and neurological symptoms of B12 deficiency, worsening the condition and delaying diagnosis, leading to irreversible morbidities [6,185]. Reynolds et al.’s comprehensive review highlighted evidence that long-term exposure to excess FA, even at doses below the upper limit (0.5–1 mg), combined with B12 deficiency, can lead to neurological harm and cognitive decline [183]. FA supplementation may increase the demand for vitamin B12, exacerbating its depletion, pernicious anemia, and associated neurological and hematological decline [186,187,188]. Following fortification mandates in countries like the United States and Australia, epidemiological studies found a link between excess folic acid and cognitive decline in older adults at risk of B12 deficiency [189,190,191,192]. However, there is still no consensus regarding the role of excess FA in potentiating neurological and cognitive impairment in individuals with a low or deficient B12 status [193,194].

A B vitamin imbalance resulting from high FA combined with inadequate B12 may lead to a functional folate deficiency, contributing to insulin resistance and gestational diabetes mellitus [195]. In pregnant rats, high FA supplementation combined with a B12-deficient diet led to oxidative stress in both the mothers and pups compared with the same supplementation with adequate B12 status [196]. Excess FA supplementation alongside B12 deficiency during pregnancy and lactation resulted in diet-dependent metabolic impairment in female rat offspring [197]. Similar studies in rats demonstrated that adequate B12 levels are required to mitigate the potential harmful effects of high FA on DNA methylation and neurodevelopment in offspring [198,199].

**Figure 2:**
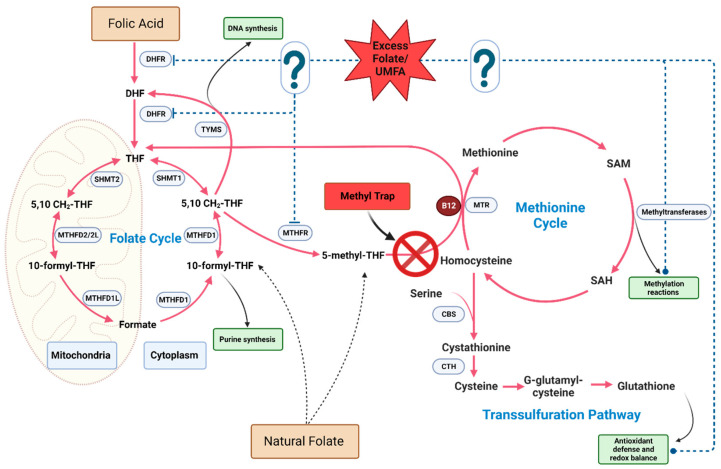
Potential biochemical impact of excess folate on one-carbon metabolism pathways. The question marks and dotted blue line represent the potential molecular targets of excess folate and/or UMFA on one-carbon meatbolic enzymes and biosynthetic outputs.

Mechanistically, the paradoxical combination of excess folate with B12 deficiency leads to a unique metabolic situation known as the methyl trap. The primary enzymatic reaction implicated in the methyl trap is catalyzed by methionine synthase (MTR), which is a vitamin B12 (cobalamin)-dependent enzyme. MTR catalyzes the conversion of homocysteine to methionine, with 5-m-THF acting as the methyl donor. During this process, 5-m-THF transfers a methyl group to cobalamin, generating methylcobalamin, which subsequently transfers the methyl group to homocysteine, yielding methionine and tetrahydrofolate (THF). THF is essential for purine and pyrimidine synthesis, which are required for DNA and RNA production. In situations of vitamin B12 deficiency, MTR activity is compromised, leading to an accumulation of 5-m-THF, as the enzyme is unable to utilize it as a methyl donor, leading to the methyl trap (Figure 2). This sequestration of folate in the form of 5-m-THF results in a functional folate deficiency, as the intracellular pool of THF is depleted. The reduced availability of THF impairs the synthesis of purines and pyrimidines, ultimately affecting DNA and RNA synthesis. Consequently, this disruption in nucleotide synthesis manifests as megaloblastic anemia, which is characterized by large, immature erythrocytes. Additionally, vitamin B12 deficiency and the subsequent impairment of MTR activity leads to an accumulation of homocysteine, as it cannot be converted to methionine. Elevated homocysteine levels are associated with an increased risk of cardiovascular disease, neurodegenerative disorders, and other adverse health outcomes.

#### 5.1.2. Accumulation of Unmetabolized Folic Acid

When folic acid intake is high, UMFA can accumulate in the bloodstream, as the body’s ability to convert folic acid to its active reduced form, 5-m-THF, in the intestine and liver becomes saturated [4,200,201]. Specifically, UMFA accumulation is associated with the saturation of the rate-limiting one-carbon enzyme DHFR. The enzymatic activation of FA by DHFR is slow in humans, leading to a persistent elevation of UMFA after limited exposure to dietary FA [201,202]. Higher levels of circulating UMFA generally correlate with increased folic acid intake [203,204]. Intakes of FA that surpass the UL are associated with an increased likelihood of having elevated UMFA levels [205,206,207,208,209]. Furthermore, a daily intake of FA in doses as low as 200 µg was found to positively correlate with circulating UMFA levels [9,200,210,211]. UMFA can act as either a competitive or non-competitive inhibitor of DHFR depending on the intracellular concentration of dihydrofolate (DHF), which is a folate cycle intermediate generated during thymidylate synthesis (Figure 2) [201]. This inhibition of DHFR can lead to the accumulation of DHF, which is a potent inhibitor of methylenetetrahydrofolate reductase (MTHFR), ultimately disrupting the one-carbon cycle [212,213,214]. As a result, essential reactions in the cycle can be dysregulated, impacting DNA synthesis and repair, as well as methylation processes. Evidence exists to support the use of UMFA as a marker for excess folate in humans, as UMFA levels generally increase in response to supplemental folic acid intake [38,215]; however, newer sensitive methods were able to detect UMFA in the majority of samples, irrespective of folic acid intake [9,216].

#### 5.1.3. Pseudo-MTHFR Deficiency

MTHFR is an enzyme that catalyzes the conversion of 5,10-methylenetetrahydrofolate to 5-m-THF, which is the biologically active form of folate. This conversion is a critical step in the folate metabolic pathway, as 5-m-THF participates in the re-methylation of homocysteine to methionine. As outlined previously, the presence of excess folic acid could competitively inhibit the enzymes involved in the conversion of other forms of folate, such as DHFR, which converts dietary folic acid to DHF and subsequently to THF. This competitive inhibition can exacerbate an existing MTHFR deficiency by further disrupting the folate metabolic pathway, leading to a functional folate deficiency despite adequate folate intake. Polymorphisms in the MTHFR gene are associated with reduced enzyme activity and increased health risks. In humans, C677T and A1298C polymorphisms are common, with an estimated 20–40% of the US population harboring one or both mutations [19].

MTHFR inhibition by excess folate is not exclusively linked to cases where genetic polymorphisms are present. In the absence of known MTHFR polymorphisms, elevated levels of folic acid resulted in a pseudo-MTHFR deficiency [68,97,217,218,219]. This leads to the disruption of the one-carbon cycle, negatively impacting methylation reactions, and potentially precipitating various health issues, including abnormal embryonic growth and neurodevelopment [218].

#### 5.1.4. Disruption of Methylation and Epigenetics

A significant portion of the negative effects linked to excess folate appeared to be associated with epigenetic alterations. The methionine cycle, which supplies one-carbon units to methylation reactions, is intricately linked to the folate cycle, with both sharing common regulatory signals. Folate is vital for the generation of S-adenosylmethionine (SAM), which is the primary methyl donor for DNA methylation.

Disturbances in folate levels arising from either low or high folate status can lead to hypo- or hypermethylation, resulting in epigenetic instability, dysregulation of gene expression, and ultimately contributing to the initiation and progression of cancer [220,221,222]. DNA hypomethylation is considered an early event in colon carcinogenesis [223]. Excess FA is capable of inducing hypomethylation in cultured human cells [224] and an alteration in methyl metabolism in mice [94,217]. Work with multiple models suggests that both folate deficiency and excess FA can disrupt the folate cycle and the connected methionine cycle, leading to dysregulated methylation [93,218,225,226]. In human lymphocytes, high folic acid intake relative to natural folate can influence DNA methylation, disrupt genomic stability, and diminish the capacity to withstand oxidative stress [21,124]. Excess folic acid supplemented to cancer cell organoids can counteract methionine dependency, thus promoting cancer stem cell formation [227]. In mice, a high-FA diet promoted hepatocellular carcinoma development through the stabilization of the methionine cycle enzyme MATIIα.

The in vivo and in vitro mechanisms through which excess maternal FA supplementation can influence neurological development were reviewed elsewhere [228], detailing behavioral, morphological, and molecular changes in the offspring’s brain exposed to FA over-supplementation. A recent investigation into some of the underlying mechanisms discovered significant changes in DNA methylation of neurodevelopmental genes, which play a crucial role in transcriptional regulation [229]. Notably, these modified epigenetic patterns persisted throughout an individual’s life, as demonstrated in the Aberdeen Folic Acid Supplementation Trial [230]. Although both animal and human studies have identified a correlation between excessive maternal FA intake and metabolic abnormalities in offspring, a clear causative link with the modified epigenetic landscape has not yet been established [60,74,75,76,231,232].

While the precise mechanisms by which excess folate impacts methylation are not clearly understood, disruption of one-carbon metabolism is the primary suspect. The prevailing theory is that excess folic acid can disrupt the balance of SAM and its demethylated product, namely, S-adenosylhomocysteine (SAH). This disruption could lead to global DNA hypomethylation, resulting in genomic instability, altered gene expression, and an increased risk of diseases. Supporting evidence is available from studies reporting that high FA levels were linked to the dysregulation of intracellular one-carbon metabolism [233], imbalance in the distribution of folate coenzymes and derivatives [218], and perturbation of methylation reactions [70,91,234,235,236,237]. Studies in mice found that excess maternal FA intake disrupted the offspring’s folate metabolism, creating an altered metabolic flux within one-carbon metabolism that mimicked folate deficiency, favoring DNA synthesis over methylation reactions [93]. Other reports confirmed that both folate deficiency and excess supplementation similarly compromised folate-dependent pathways [238]. In a zebrafish model, excessive FA led to abnormal cardiac development and defects in folate metabolism similar to folate depletion using the antifolate drug methotrexate [126]. Both excess paternal FA supplementation and FA depletion in mice resulted in similar deleterious transgenerational outcomes that potentially stem from altered methylation of imprinted sperm genes [64].

The literature presents compelling evidence that excess folate disrupts one-carbon metabolism, significantly impacting methylation reactions. It remains unclear whether the resulting epigenetic changes are caused solely by the direct disruption of one-carbon metabolism by excess FA or if other unidentified pathways are involved.

### 5.2. One-Carbon-Metabolism-Independent Mechanisms

A significant limitation to previously discussed reports hypothesizing that UMFA could be the driver behind the effects of excess folate is that UMFA is not known to accumulate in biological tissues and inside cellular compartments. Unlike reduced folate, UMFA has a low affinity for the reduced folate carrier transporter (RFC) and is not a substrate for the enzyme folate polyglutamate synthase (FPGS) [239,240]. UMFA cannot directly participate in one-carbon cycle metabolism and binds weekly to folate enzymes. However, UMFA can bind to and be transported into tissues by the folate receptor (FOLR) and the proton-coupled folate transporters (PCFTs). This prompts an inquiry into whether the effects of excess folate on one-carbon metabolism, as observed across multiple studies, arise from UMFA’s direct interactions with one-carbon enzymes, indirectly by increasing total systemic folate, or through alternate pathways independently of one-carbon metabolism. The following studies detail the effects of excess FA and natural folate that are mediated via non-canonical pathways.

Folic acid could operate through mechanisms different from natural folate. One notable mechanism concerns the folate receptor (FOLR) binding to FA, facilitating its transport, and potentially activating downstream effectors. Folate receptors have a much higher affinity to FA compared with natural folates. FOLRα facilitates the intake of FA through receptor-meditated endocytosis. FOLRα is ubiquitously expressed in the epithelia of normal tissues (kidney, placenta, blood–brain barrier), as well as being overexpressed in many cancers, such as ovarian, breast, and colon cancers [241]. The role of FOLRα in mediating tumorigenesis and the associated physiological function in cancer are not clear [158,242]. In the presence of FA, FOLRα could translocate into the nucleus and function as a transcription factor, impacting the genes responsible for cellular pluripotency [203,204]. FA-bound FOLR acts as a signaling molecule that activates downstream targets, such as the pro-oncogene STAT3, extracellular signal-regulated kinase (ERK) kinases, and cellular Sarcoma (SRC) pathways [205,206,207,208,209].

Unlike natural folate, folic acid is linked to the regulation of neurogenesis in the ventral hippocampus in rats [243]. A study in piglets found that a high-FA diet altered the expression of liver proteins involved in oxidative responses, metabolic regulation, and cancer pathways compared with a control diet (30 mg FA/kg vs 1.3 mg FA/kg diet) [244]. Excess periconceptual FA led to upregulated Fos expression in mice [91] and activation of β-catenin (Wnt pathway) in the brain of male mouse offspring [98]. In studies that compared the effects of supplementing excess synthetic and natural folate in rats, the authors observed contrasting metabolic shifts leading to weight gain, leptin dysregulation, and increased food intake in the female offspring when dams were fed excess 5-m-THF compared with excess FA, with FA significantly impacting neurotransmitter signaling genes [173,245]. In a prostate cancer model, folate was found to be directly involved in oncogenic signaling pathways, specifically the PI3K-AKT-mTOR cascade [246]. Similarly, in a colon carcinogenesis mouse model, a significant increase in mTOR signaling was recorded in mice supplemented with folic acid compared with those without supplementation [20]. In addition to the observed effects on gene expression, FA was found to impact posttranslational modifications and protein–protein interaction [241]. These findings highlight an extensive interplay between folate intake and regulatory processes that mediate gene expression and protein interactions that require further research and analysis.

## 6. Folate: A Regulator of Organismal Aging?

Recently, the one-carbon cycle has been identified as a central regulator of organismal aging [247,248]. Metabolomic analysis of long-lived *C. elegans* and mouse models revealed common signatures and regulation of the one-carbon folate cycle. In a robust analysis, Annibal et al. demonstrated that supplementing long-lived *C. elegans* mutants with physiological levels of folate partially abrogated the lifespan extension, while RNAi knockdown of DHFR extended it in wild-type worms [247]. Our preliminary work confirmed these reports; we observed increased longevity in C57/Bl6 mice on a folic-acid-restricted diet compared with those receiving a folic-acid-supplemented diet. Furthermore, exposing *C. elegans* to the DHFR inhibitor methotrexate significantly increased the worm’s lifespan, while supplementing the media with folate shortened it in a dose-dependent manner. These findings highlight the folate cycle as a shared causal mechanism for longevity and add to the urgency for further research.

## 7. Limitations

The expansive domain of nutrition research focusing on folate’s impact on health has witnessed significant diversity and evolution over the last three decades. Historically, a vast majority of studies emphasized the dangers of folate deficiency and the benefits of maintaining adequate folate intake. This predominant focus led to a relative sidelining of the potential ramifications of excessive folate consumption, which is an issue gaining relevance due to escalating folic acid intake in contemporary diets. However, a surge in recent literature shed light on the potential deleterious effects associated with high folate intake. The increasing volume and frequency of these publications prompted a re-evaluation of longstanding paradigms related to folate intake and health.

Given the breadth and depth of this field, constructing a comprehensive review encompassing every nuance, controversy, and equivocal finding is inherently daunting. Hence, this review did not intend to serve as an exhaustive or entirely impartial examination of the folate research spectrum. Instead, its primary objective lay in accentuating the emergent findings and mechanisms that robustly correlate excessive folate consumption with health effects and the etiology of diseases. Nevertheless, efforts were made to ensure a balanced discourse, underscore prevailing controversies, and highlight areas necessitating further investigation.

Caution must be exercised when interpreting the results from folate studies conducted in preclinical models. Rodents display significant and measurable differences in folate requirements, metabolism, and the activity of rate limiting one-carbon enzymes [201]. Rats and mice blood folate levels are on average 6-8 fold higher than humans [157]. This observation should lead to careful consideration when extrapolating rodent data to human disease. However, the phenotypes induced by excess folate in rodents can undoubtedly provide valuable insights into the risks and mechanisms involved. 

## 8. Conclusions

As researchers, including ourselves, strive to unravel the conserved and intricate dynamics governing the relationship between folate, health, disease, and longevity, a crucial question arises: could exposing the human population to excessive levels of folate potentially accelerate aging and increase the risk of disease?

The key to answering this question lies in identifying causal underlying mechanisms that can robustly link excess folate intake to the observed detrimental effects. Achieving this goal requires substantial research efforts and collaboration across multiple scientific disciplines to identify and address the existing knowledge gaps. The importance of pursuing such research cannot be overstated, as these gaps in knowledge may be unknowingly and insidiously compromising the health of hundreds of millions of people worldwide who are consuming excess folate.

## Data Availability

Not applicable.
